# Determination of phosphate in soil extracts in the field: A green chemistry enzymatic method

**DOI:** 10.1016/j.mex.2015.04.003

**Published:** 2015-04-22

**Authors:** Ellen R. Campbell, Kayla Warsko, Anna-Marie Davidson, Wilbur H. (Bill) Campbell

**Affiliations:** NECi, Lake Linden, MI 49945, USA

**Keywords:** HEPES, 4-(2-hydroxyethyl)-1-piperazineethanesulfonic acid, IMAC, immobilized metal-ion affinity chromatography, MESG, 2-amino-6-mercapto-7-methylpurine ribonucleoside, PNP, purine nucleoside phosphorylase (EC 2.4.2.1), Soil phosphate determination in the field, Soil phosphate, Soil phosphorus, Field, Enzymatic, Green chemistry, Open-source portable photometer

## Abstract

Measurement of ortho-phosphate in soil extracts usually involves sending dried samples of soil to a laboratory for analysis and waiting several weeks for the results. Phosphate determination methods often involve use of strong acids, heavy metals, and organic dyes. To overcome limitations of this approach, we have developed a phosphate determination method which can be carried out in the field to obtain results on the spot.

This new method uses:

• Small volumes.
• An enzymatic reaction.
• Green chemistry.

First, the soil sample is extracted with deionized water and filtered. Next, an aliquot of the soil extract (0.5 mL) is transferred to a disposable cuvette, containing 0.5 mL of reaction mixture [200 mM HEPES, pH 7.6, 20 mM MgCl_2_, with 80 nmol 2-amino-6-mercapto-7-methylpurine ribonucleoside (MESG) and 1 unit of recombinant purine nucleoside phosphorylase (PNP; EC 2.4.2.1)], mixed, and incubated for 10 min at field temperature. Absorbance of the completed reaction is measured at 360 nm in open-source, portable photometer linked by bluetooth to a smartphone. The phosphate and phosphorus content of the soil is determined by comparison of its absorbance at 360 nm to a previously prepared standard phosphate curve, which is stored in the smartphone app.

## Graphical abstract

**Figure:**
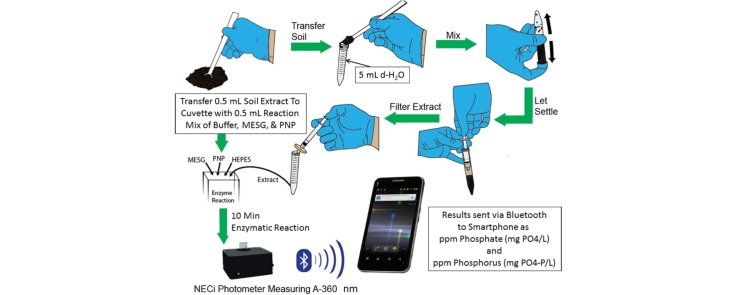

## Method details

In the field soil phosphate analysis method, samples are taken in the field using a clean small scoop or spatula. For reproducibility, the soil samples should be about the same volume. The soil samples are transferred to 15 mL Falcon™ tubes with caps, which are labeled and contain 5 mL of deionized water (15 mL BD Falcon™ Centrifuge Tubes, polypropylene, or equivalent). The sample tubes are capped and inverted 10 times with shaking and allowed to settle for 15 min. The liquid in the sample tube along with some soil is transferred to a 10 mL syringe, which is subsequently fitted with a filter (B-D™ Disposable Syringes, Luer-Lock Tips, 10 mL, # 14823 2A; Cole-Parmer Nylon Syringe Filters, 0.45 μm, 25 mm diameter; Item# UX-02915-14; equivalent syringes and filters can be used). The soil extract is injected via the filter into another labeled 15 mL Falcon™ tube. Labeled reaction microfuge tubes (BrandTech clear 1.5 mL non-sterile disposable microcentrifuge tubes with lids, or equivalent) are set up with 0.5 mL of a reaction mixture containing: 200 mM HEPES, pH 7.6, 20 mM MgCl_2_, containing 80 nmol MESG (http://www.berryassoc.com/prodetails.asp?product_number=PR%203790) and 1 unit of recombinant PNP (NECi recombinant PNP1, 1 unit = 1 μmol phosphate consumed per min, see Nitrate.com; or equivalent); which has been allowed to come field temperature. A 500 μL sample of each soil extract is transferred by micropipette to an appropriately labeled reaction microfuge tube containing reaction mixture, the tube capped, and inverted 3 times. The reaction tubes are incubated for about 10 min at field temperature; exact timing is not important since the reaction goes to completion. The contents of the reaction tubes are transferred to methylacrylate (PMMA) disposable cuvettes (UV-Cuvette Disposable Photometer Cuvette, VWR catalog No. 47727-024, or equivalent) and the absorbance at 360 nm is measured for each soil sample and a deionized water blank using a portable photometer. The absorbance at 360 nm for each soil extract sample is compared to a standard curve prepared in advance with certified KH_2_PO_4_ standard 1000 ppm (LabChem Inc., www.labchem.net, catalog No. LC 18570-1) diluted in deionized water to the range of 0.00–5.00 ppm (mg PO_4_/L). The reagent blank (i.e., 0 ppm) 360 nm absorbance is subtracted from the absorbance for each standard and sample to obtain the net 360 nm absorbance, which is plotted versus the nominal phosphate concentration of the standards. Using the linear regression equation of the standard curve, the inorganic phosphate content of each soil sample is calculated and recorded. The results may be reported as ppm phosphate per volume of soil sampled (i.e., volume of the scoop used to sample the soil); or mg phosphate per liter per weight soil sample. The results may also be reported as phosphorus, by simply dividing the phosphate results by 3.1 to obtain ppm phosphorus (mg PO_4_–P/L). The ratio of phosphate to phosphorus of 3.1 is simply the division of the formula weight of H_2_PO_4_^−^ by elemental weight of P (97/31 = 3.1). While this method gives good estimates of inorganic or available phosphate in soil samples, for greater precision, the soil should be dried to constant weight and 1 gm of dry soil extracted with 5 mL of deionized water.

## Methods to reduce turbidity

1. Filter the supernatant using Millipore 0.45 μ filter with a Durapore^®^ membrane disposable filter. Use filtered extract as soil sample in assay. OR
2. Centrifuge samples for 5 min at 1500 × *g*. Use centrifuged supernatant as soil sample in assay.
3. Pipet 500 μL of reaction mixture into Perfector Scientific methacrylate 1.5 mL cuvettes.
4. Pipet 500 μL of standards and clarified soil extracts into labeled cuvettes.
5. Invert capped cuvette several times to mix and incubate for 10 min.
6. Blank spectrophotomer using deionized water @ 360 nm, and measure absorbance of standard and samples @ 360 nm.
7. Subtract the absorbance at 360 nm of the reagent blank (i.e., 0 ppm phosphate standard) from the absorbance @ 360 nm of the standards and samples to obtain the net absorbance.

## Development of the phosphate test for soil extracts

**Fig. 1:**
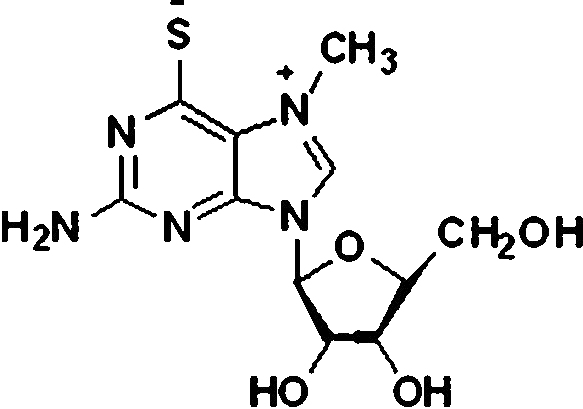
Structure of MESG.

To formulate a simple and easy to use method for determining ortho-phosphate in soil extracts by an enzymatic reaction with small volumes and green chemistry, we chose purine nucleoside phosphorylase (PNP; EC 2.4.2.1) and the artificial substrate 2-amino-6-mercapto-7-methylpurine ribonucleoside (MESG; see Fig. 1). The deoD gene sequence from *Escherichia coli* coding for the PNP enzyme (Genbank accession No. M60917.1), which had been previously expressed in *E. coli* [6], was expressed in *E. coli* with an N-terminal His-tag and purified by IMAC [2]. In the presence of inorganic phosphate (H_2_PO_4_^−^), PNP catalyzes the conversion of MESG to 2-amino-6-mercapto-7-methyl purine and ribose-1-phosphate. The purine product yields an increase in absorbance at 360 nm in equal molar ratio to the inorganic phosphate in the reaction mixture. This enzymatic reaction was designed to measure PNP activity and quantify phosphate in biological samples a number of years ago [9], [10], [3]). It has been implemented in many commercial kits for determining inorganic phosphate. Next we developed a simple way to extract soil in deionized water where a sample of soil was mixed with water and filtered using a syringe method. We also compared centrifugation of the soil extract sample to the filtration method to determine which of the two methods was better in terms of giving more reproducible results.

## Standard curve for the enzymatic test for phosphate in soil

**Fig. 2:**
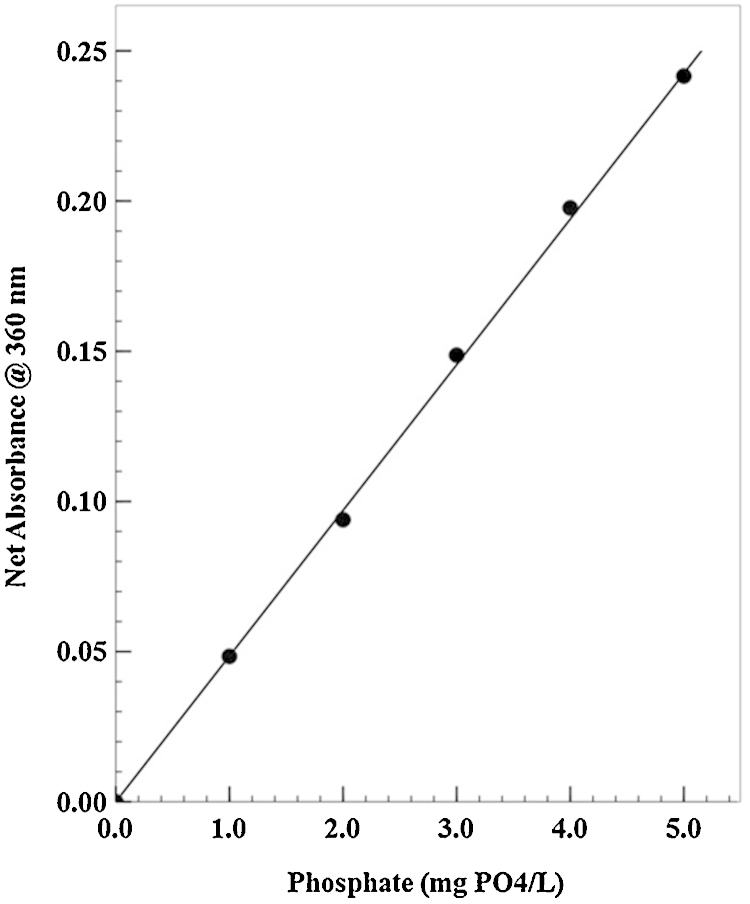
Typical phosphate standard curve for field soil phosphate test. The equation of the liner regression line fitted to the data set is: *Y* = 0.0489 *X* − 0.0005 Correlation coefficient (*r*^2^) = 0.9993. The standard curve can be converted to measure phosphate–phosphorus by dividing the phosphate concentration by a factor of 3.1 to yield mg phosphate–phosphorus per liter (mg PO_4_–P/L). When this is done with the data shown in the figure, the slope (*x*) = 0.0151 mg PO_4_–P per L, while the intercept and correlation coefficient do not change.

A set of phosphate standards was prepared by diluting a certified 1000 ppm (1 mg/mL) potassium phosphate standard (KH_2_PO_4_; Lab Chem Inc., Catalog No. LC 18570-1), to encompass the range of interest (i.e., 1.00–5.00 mg phosphate/L). The phosphate standards were analyzed by the PNP catalyzed reaction with MESG and the absorbance at 360 nm plotted versus the nominal phosphate concentration of standards (see Fig. 2). After the background absorbance of the reagent blank is subtracted, the standard curve is linear with a correlation coefficient = 0.999. Using the equation of the fitted line, the amount of phosphate extracted from the soil sample is easily determined. The phosphate content (mg PO_4_/L) can be converted to phosphorus content by dividing by 3.1 (mg PO_4_–P/L). Furthermore, the phosphate–phosphorus content of the soil can be converted to the commonly used units of mg phosphorus per kg soil by multiplying by a factor of 5, since 1 g of soil is extracted and 0.5 mL of deionized water extract is analyzed.

## Verifying the validity of the field soil phosphate test

Certified soil samples with known phosphate content were obtained from Prof. R.O. Miller, Colorado State Univ., Fort Collins Co., extracted and analyzed using the field method. The results of the comparison of the field method phosphate determination to known phosphate content of soils is shown in Table 1. From these results, the field soil phosphate test method gives results equivalent to those obtained by a standard method.

## Comparison of methods to reduce turbidity of soil extracts in the field

Two methods to reduce turbidity are available: filtration and centrifugation. We compared these two methods using the field soil phosphate test method and local soil samples (Table 2). The two methods give equivalent results, but the filtration method had a lower standard deviation than the centrifugation method for the soil extracts analyzed. Furthermore, the filtration method is easier to implement in the field since it does not require a source of 120 V power and an expensive micro-centrifuge. To further validate the filtration method for the field phosphate test method, we carried out a standard addition study with the same soil samples (Table 3). The recovery of the phosphate spike added to each soil extract was within ±10% of the amount of phosphate spike added. These results confirm the effectiveness of the filtration method for implementing the field soil phosphate test.

## Practical aspects of the field soil phosphate method

Extraction of soil for determination of phosphate is a science unto itself; see for example Pierzynski [7], and Abdu [1]. Soil varies in the amount of moisture present when sampled in the field. Thus, soil samples are usually dried to constant weight prior to analysis for phosphate content and a specific weight of soil taken for analysis [4]. To circumvent this barrier, a small scoop is used to take a specific volume of soil for the field test. This approach clearly has limitations. In order for the field soil phosphate test to be practical for on-site estimates of phosphate content, the moisture content of the set of samples is assumed to be similar. Once the phosphate content of the set of soil samples has been determined with the field soil phosphate test, the user (a crop consultant for example) can easily estimate the amount of phosphate fertilizer which needs to be applied to the crop plant field to bring up the phosphate available to the plants to maximize yield. Waiting for analytical laboratory results means time lost when the crops could be growing with optimum phosphate available. With the field soil phosphate test results, the user (crop consultant) can immediately apply the correct amount of phosphate fertilizer with less risk of over fertilizing; thus, avoiding wasted expensive fertilizer and runoff from the field polluting nearby waterways.

## Portable photometer wirelessly interfaced to a tablet PC

**Fig. 3:**
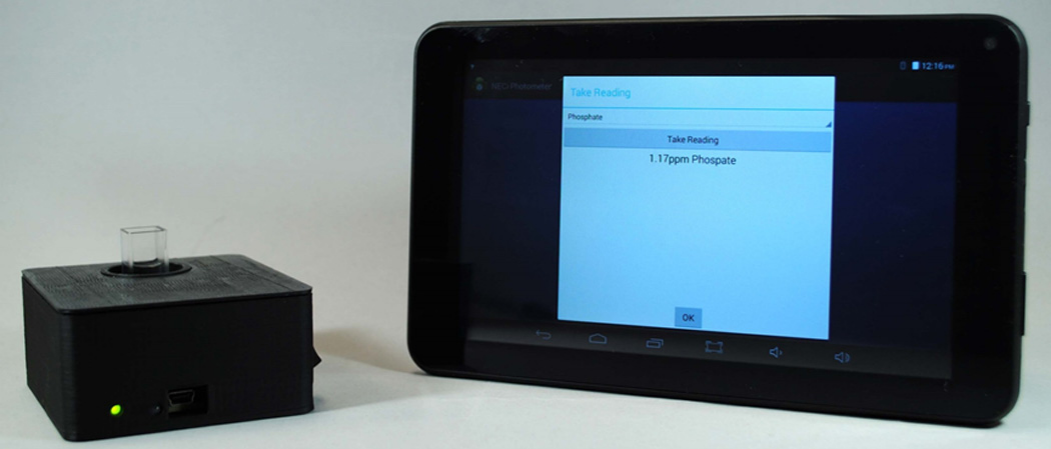
NECi open-source portable photometer with tablet PC running the NECi phosphate soil analysis app. The photometer is shown with a 1.5 mL disposable cuvette installed, which is 1 cm in path length, which provides a measure of dimensions of the photometer (8 × 7.2 ×3.5 cm). The NECi photometer is connected to the table PC by bluetooth prior to taking readings. The setting menu allows the user to blank the photometer prior to taking readings.

**Fig. 4:**
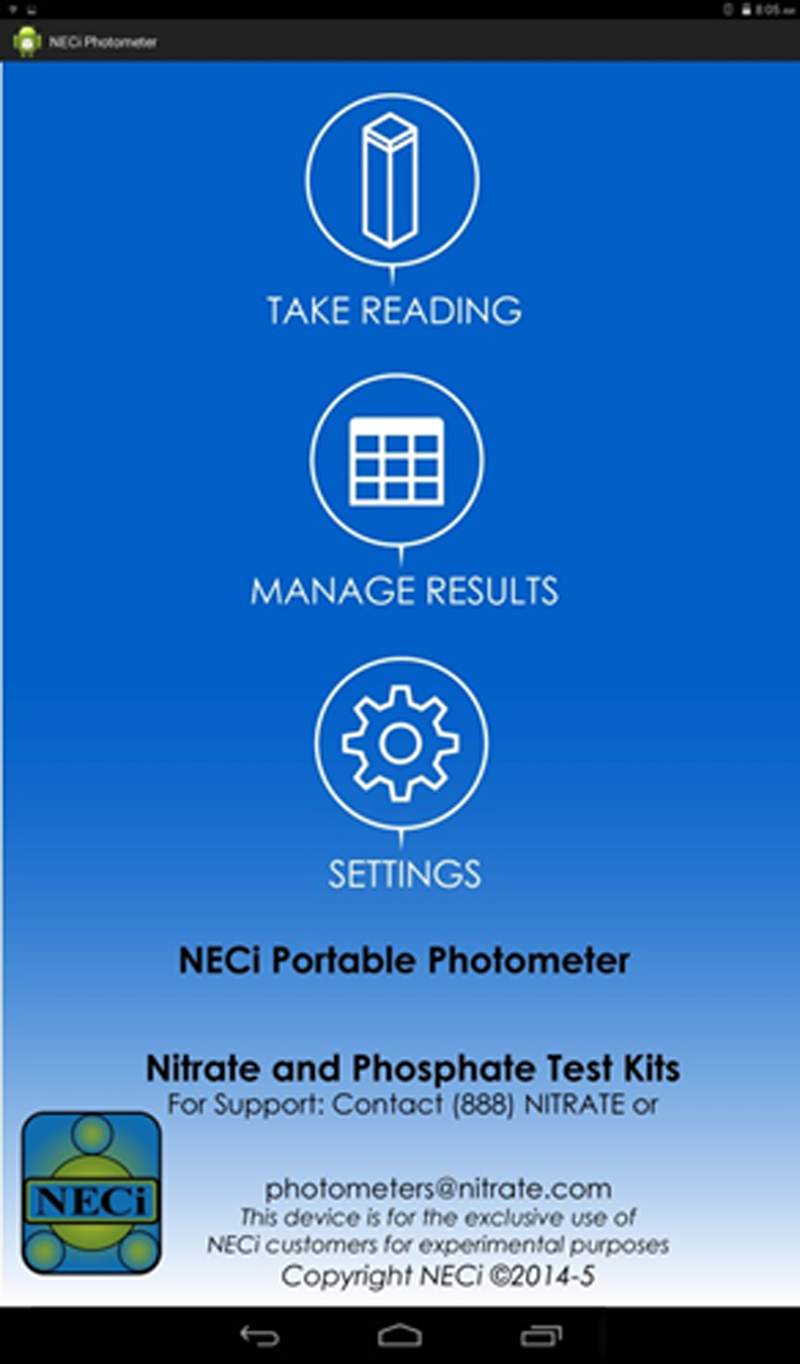
Home screen for smart phone or tablet app. The user can simply read the absorbance at 360 nm for each standard and soil extract sample and record them by hand. Alternatively, the user can set up a list of standards and samples and store them along with the absorbance at 360 nm. The GPS function of a smart phone can be used to establish the location where the soil samples were taken and analyzed, which can be also be saved. The list and data can be saved in the cloud and accessed at a later time using a PC via the internet.

Another consideration is the availability of a portable photometer which can be used for measuring absorbance at 360 nm, since this wavelength is not visible it cannot be estimated by eye. To overcome this limitation, we have developed an inexpensive open-source portable photometer capable of measurements at 360 nm: NECi Photometer and software app for smart phone/tablet PC (Fig. 3). The home screen for the smart phone and tablet PC app is shown in Fig. 4. The NECi Field Photometer is perfectly suited to the small volume of the field soil phosphate test and associated smartphone or table PC automatically reports the phosphate content of the sample in mg phosphate/L. The NECi Field Photometer is currently being tested and will be available soon. Other portable colorimeters can be used, if they accept the small cuvette used here.

## Interferences

Finally, are there inferences with the field soil phosphate test? Constituents of soil commonly found include metal ions such as calcium, magnesium, sodium, iron, and sulfate and chloride. These were tested in the phosphate assay and none interfered when present at low levels (1–10 mM). However, if salt (NaCl) is used to extract the soil or is present in the sample (seawater for example), then a comparable amount must be added to the standards when doing the standard curve. The only known substance which interferes with the test is arsenate, an analog of phosphate. Arsenate is an alternate substrate for *E. coli* PNP [5]. Since many soils in the U.S. contain arsenic at ppb levels [8], free arsenate content of soils is expected to be too low to significantly interfere with the field soil phosphate test; thus, this is not a serious problem. The field soil phosphate test can be used without fear of obtaining false positive or negative results.

## Alternate application of the enzymatic phosphate analysis method to water

To illustrate the application to environmental samples, the enzymatic inorganic phosphate method with green chemistry described herein has also been employed to analyze water samples. A set of inorganic phosphate standards with a range from 5 to 50 mg Phosphate per L, was used for establishing the standard curve with 100 μL sample size in a 1.0 mL assay final volume. Each standard was analyzed 6 times and the mean absorbance at 360 nm (corrected for background absorbance 360 nm) was plotted versus the nominal inorganic phosphate concentration. These data were fitted with linear regression and the equation of the fitted line was:

$$\text{Inorganic phosphate concentration (mg}{PO}_{4}/L)=\frac{\left(\text{A-}360-0.0007\right)}{0.00979}$$

The standard curve equation was used to calculate the inorganic phosphate concentration and converted to phosphate phosphorus (mg PO_4_–P/L), in Lake Linden, MI, tap water; two wastewater samples collected from a waste treatment plant in Denver, CO; and a wastewater sample collected from a paper mill in Michigan (Table 4). The samples were each analyzed 4 times and the mean value and standard deviation for inorganic phosphate content, in mg PO_4_/L, was calculated using the equation of the standard curve. The values obtained were converted to mg phosphate–phosphorus (PO_4_–P) by dividing by 3.1 (the ratio of the molecular weight of phosphate to phosphorus). These water samples had also been analyzed for nitrate–N (mg NO_3_–N/L) content in a study carried out for validation of the NECi enzymatic nitrate determination method conducted for the Environmental Protection Agency (US EPA), which will be published in the near future. The nitrate–N content of the samples is also presented in Table 4. As shown in Table 4, local tap water has a low content of both phosphate and nitrate. In comparing the two samples from the Denver area wastewater treatment plant, the process substantially removed the nitrate by ∼95%, while phosphate was reduced by only about 30%. The paper mill wastewater contained a substantial level of phosphate, but was very low in nitrate content. Thus, the NECi green chemistry enzymatic phosphate analysis method is useful for on-site evaluation of environmental water samples including lakes, streams, rivers, and seawater.

## Figures and Tables

**Table 1:** Comparison of phosphate–phosphorus content of filtered deionized water extracted certified soil by ALP standard method and field soil phosphate method.

Soil sample^a^	Certified phosphate–P (mg PO_4_–P per Kg soil)	Field soil phosphate–P test (mg PO_4_–P per Kg soil)	RPD^b^ (%)
1	6.50	6.40	1.53
2	4.10	3.94	3.98

a Soil sample 1 = SRS-1412 BW-OK; Soil sample 2 = SRS-1413 USI-IL; certified phosphate–P values are the median value for standard reference soil (SRS) determined by 85 laboratories participating in Agricultural Laboratory Proficiency (ALP) Program in 2014 (http://www.collaborativetesting.com/801-Soil-Analyses.aspx?DepartmentId=40).

b Relative percent difference = 100 × (certified P − field P)/((certified P + field P)/2).

**Table 2:** Field soil phosphate analysis for inorganic phosphate by two procedures.

Sample ID	Description	Filtered^a^	Centrifuged^a^	RPD^b^
		Phosphate (mg/L)	Phosphate (mg/L)	(%)
1	Home near bird feeder	2.66 ± 0.17	2.74 ± 0.34	2.9
2	NECi garden	3.05 ± 0.18	2.93 ± 0.43	4.0
3	Calumet MI public school	1.27 ± 0.09	1.29 ± 0.14	1.3

a Soil samples were either filtered or centrifuged and 4 replicates analyzed with the mean and standard deviation.

b Relative percent difference = 100 × (centrifuged − filtered)/((centrifuged + filtered)/2).

**Table 3:** Field soil phosphate analysis of spiked soil extracts by filter protocol.^a^

Sample ID	Description	Filtered	Spiked (+5 mg/L)	Difference	Recovery
		Phosphate (mg/L)	Phosphate (mg/L)	Phosphate (mg/L)	(%)
1	Home near bird feeder	2.7	8.0	5.3	106
2	NECi garden	3.1	8.0	4.9	98
3	Calumet MI public school	1.3	6.0	4.7	94

a A standard phosphate curve from 1 to 10 mg phosphate/L was used.

**Table 4:** Inorganic phosphate–P and nitrate–N content of water samples.

Sample	Description	Phosphate–P^a^ (mg/L)	Nitrate–N^b^ (mg/L)
DW1	Lake Linden, MI tap water	0.81 ± 0.01	0.32 ± 0.01
WW2	Denver area treatment plant wastewater effluent #1	5.42 ± 0.11	4.07 ± 0.01
WW3	Denver area treatment plant wastewater effluent #2	3.74 ± 0.05	0.23 ± 0.01
WW4	Michigan paper mill waste stream effluent	3.82 ± 0.17	0.040 ± 0.001

a Field water phosphate test of 4 replicates with mean and standard deviation. DW1 was analyzed with low range phosphate standard curve (0–5 ppm).

b NECi Enzymatic nitrate analysis method implemented with discrete analyzers in 12 laboratories with mean and standard deviation.
